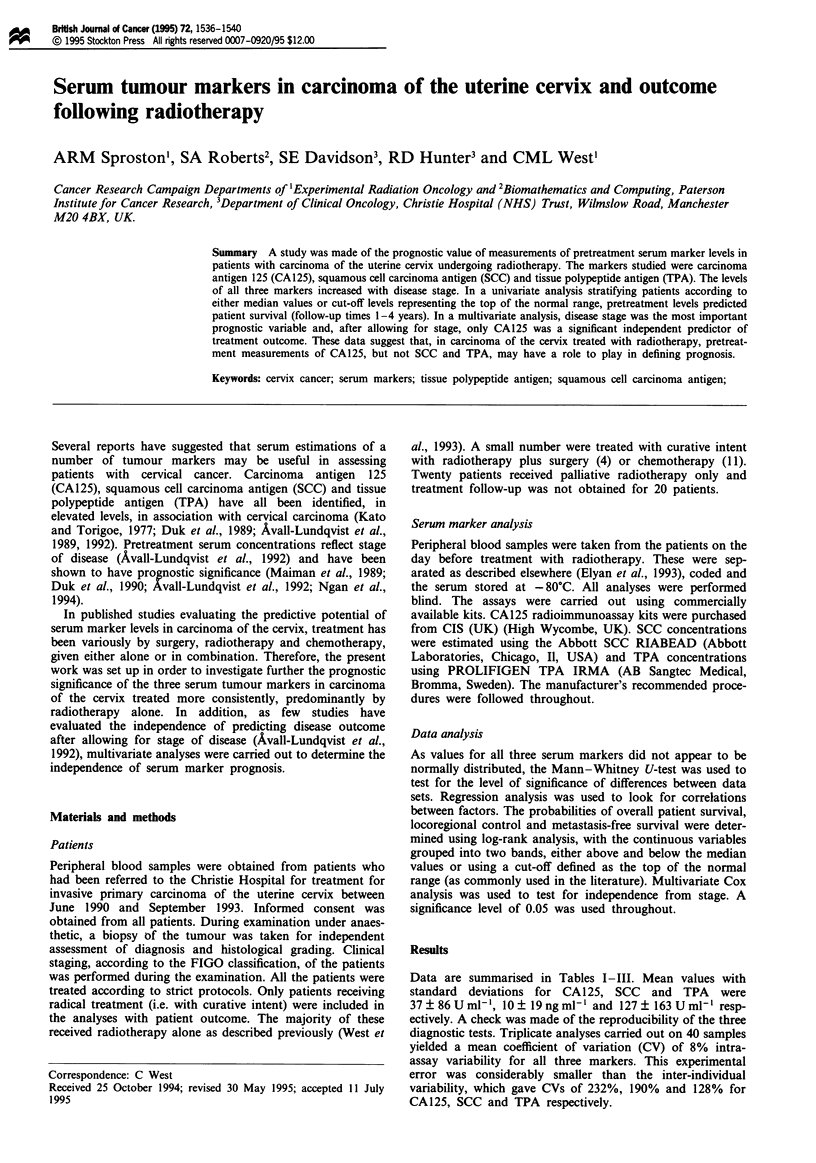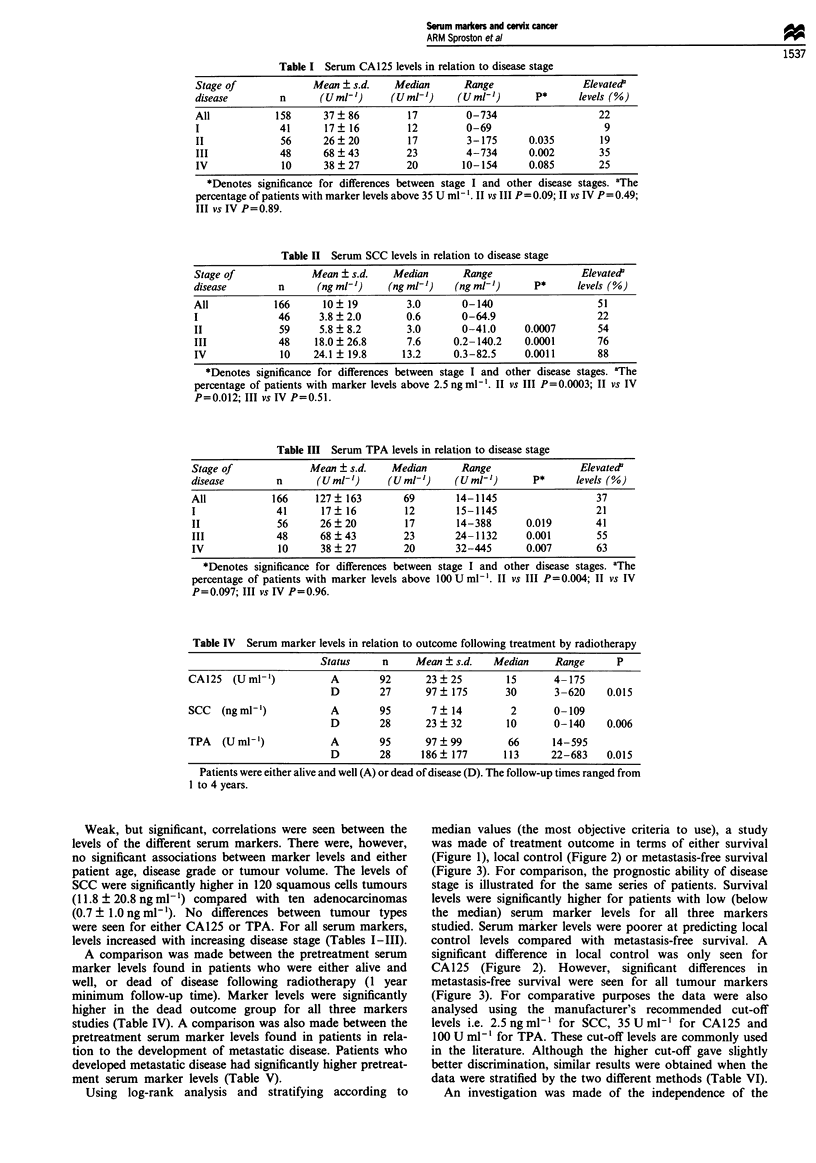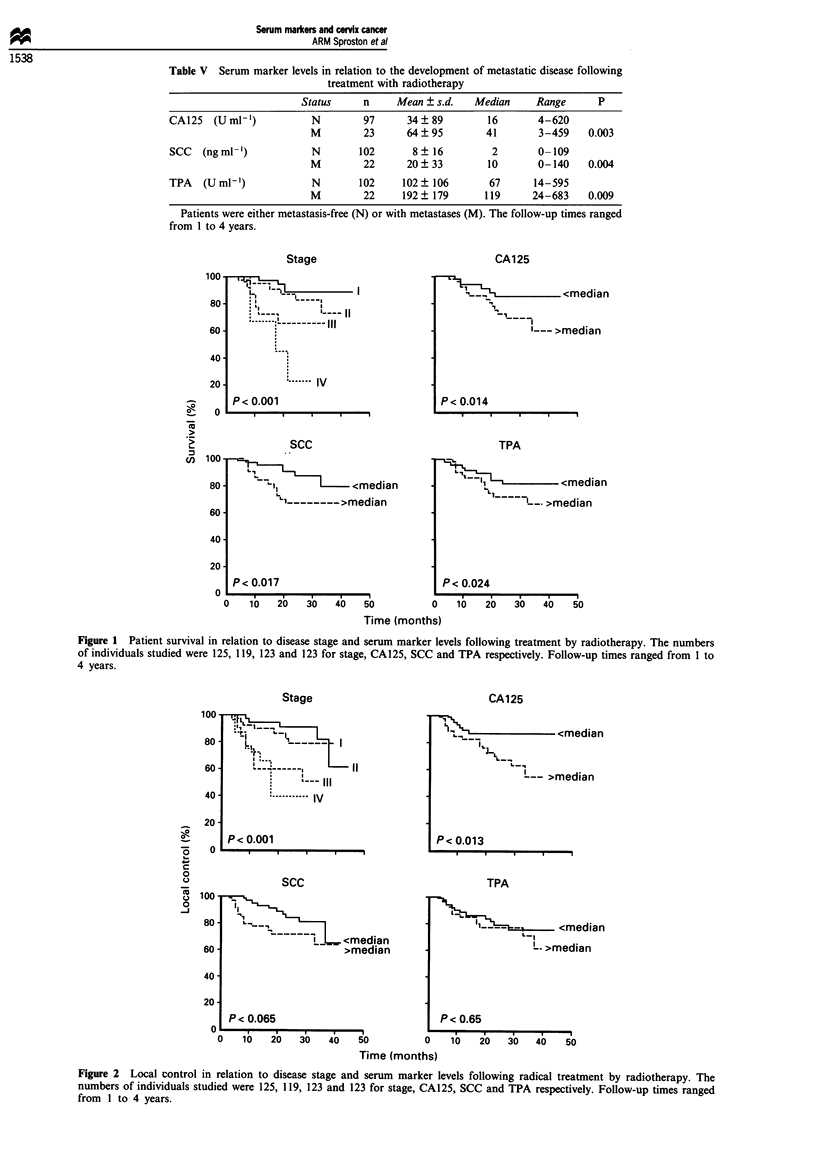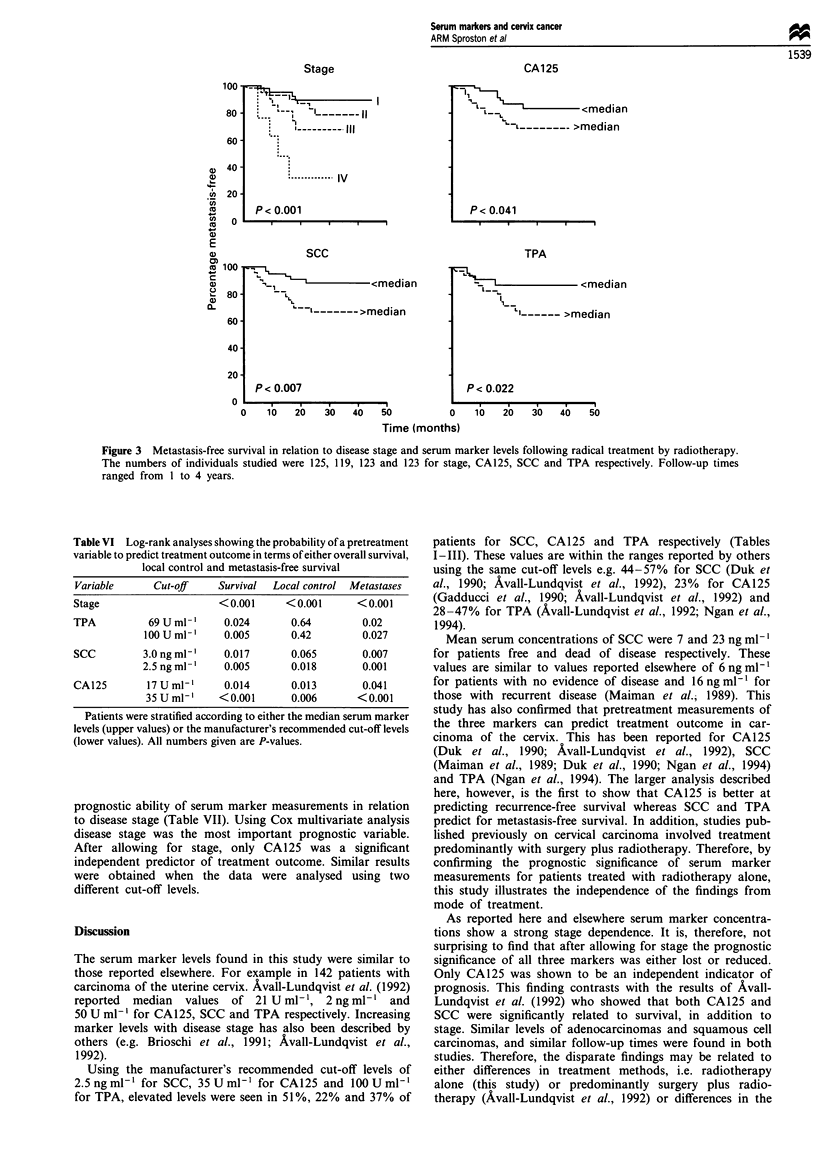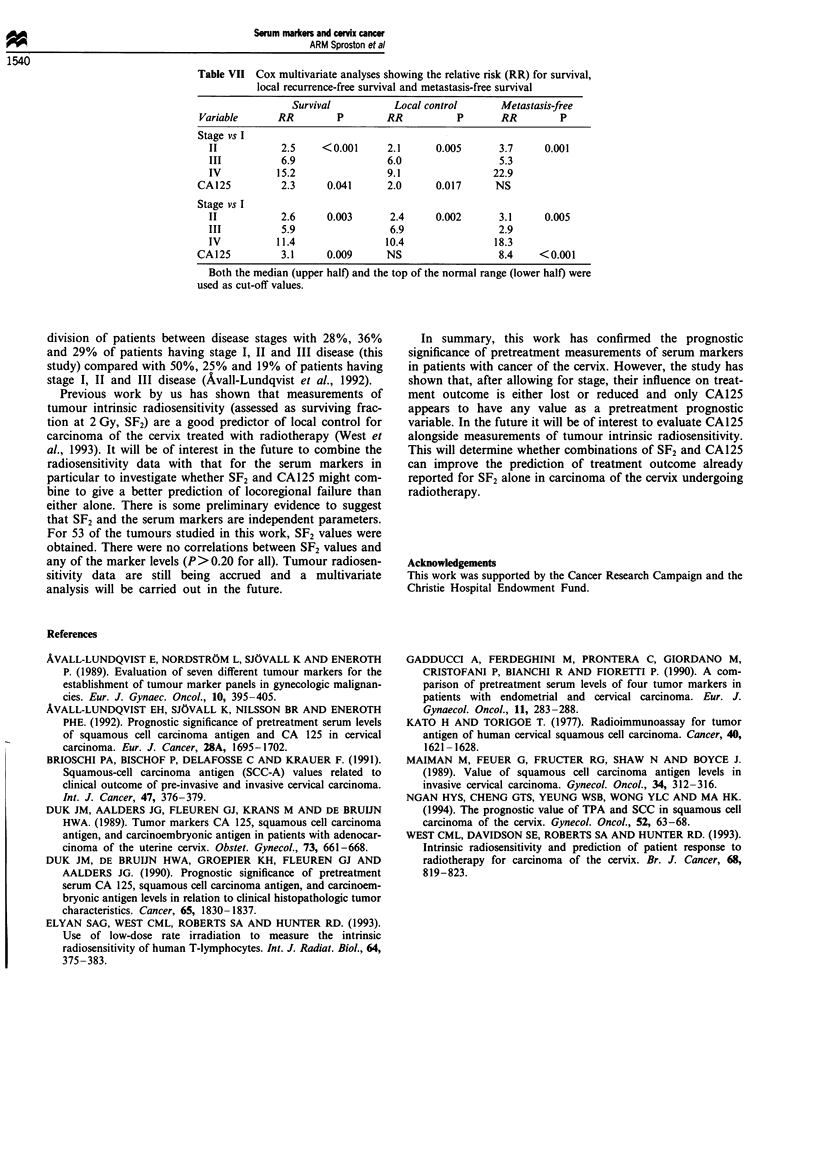# Serum tumour markers in carcinoma of the uterine cervix and outcome following radiotherapy.

**DOI:** 10.1038/bjc.1995.543

**Published:** 1995-12

**Authors:** A. R. Sproston, S. A. Roberts, S. E. Davidson, R. D. Hunter, C. M. West

**Affiliations:** Cancer Research Campaign Department of Experimental Radiation Oncology, Paterson Institute for Cancer Research, Manchester, UK.

## Abstract

A study was made of the prognostic value of measurements of pretreatment serum marker levels in patients with carcinoma of the uterine cervix undergoing radiotherapy. The markers studied were carcinoma antigen 125 (CA125), squamous cell carcinoma antigen (SCC) and tissue polypeptide antigen (TPA). The levels of all three markers increased with disease stage. In a univariate analysis stratifying patients according to either median values or cut-off levels representing the top of the normal range, pretreatment levels predicted patient survival (follow-up times 1-4 years). In a multivariate analysis, disease stage was the most important prognostic variable and, after allowing for stage, only CA125 was a significant independent predictor of treatment outcome. These data suggest that, in carcinoma of the cervix treated with radiotherapy, pretreatment measurements of CA125, but not SCC and TPA, may have a role to play in defining prognosis.


British Journal of Cancer (1995) 72, 1536–1540
© 1995 Stockton Press All rights reserved 0007-0920/95 $12.00

### Serum tumour markers in carcinoma of the uterine cervix and outcome following radiotherapy

ARM Sproston[1], SA Roberts[2], SE Davidson[3], RD Hunter[3] and CML West[1]

*Cancer Research Campaign Departments of [1]Experimental Radiation Oncology and [2]Biomathematics and Computing, Paterson Institute for Cancer Research, [3]Department of Clinical Oncology, Christie Hospital (NHS) Trust, Wilmslow Road, Manchester M20 4BX, UK.*

**Summary** A study was made of the prognostic value of measurements of pretreatment serum marker levels in patients with carcinoma of the uterine cervix undergoing radiotherapy. The markers studied were carcinoma antigen 125 (CA125), squamous cell carcinoma antigen (SCC) and tissue polypeptide antigen (TPA). The levels of all three markers increased with disease stage. In a univariate analysis stratifying patients according to either median values or cut-off levels representing the top of the normal range, pretreatment levels predicted patient survival (follow-up times 1–4 years). In a multivariate analysis, disease stage was the most important prognostic variable and, after allowing for stage, only CA125 was a significant independent predictor of treatment outcome. These data suggest that, in carcinoma of the cervix treated with radiotherapy, pretreatment measurements of CA125, but not SCC and TPA, may have a role to play in defining prognosis.

**Keywords:** cervix cancer; serum markers; tissue polypeptide antigen; squamous cell carcinoma antigen;

Several reports have suggested that serum estimations of a number of tumour markers may be useful in assessing patients with cervical cancer. Carcinoma antigen 125 (CA125), squamous cell carcinoma antigen (SCC) and tissue polypeptide antigen (TPA) have all been identified, in elevated levels, in association with cervical carcinoma (Kato and Torigoe, 1977; Duk *et al.*, 1989; Åvall-Lundqvist *et al.*, 1989, 1992). Pretreatment serum concentrations reflect stage of disease (Åvall-Lundqvist *et al.*, 1992) and have been shown to have prognostic significance (Maiman *et al.*, 1989; Duk *et al.*, 1990; Åvall-Lundqvist *et al.*, 1992; Ngan *et al.*, 1994).

In published studies evaluating the predictive potential of serum marker levels in carcinoma of the cervix, treatment has been variously by surgery, radiotherapy and chemotherapy, given either alone or in combination. Therefore, the present work was set up in order to investigate further the prognostic significance of the three serum tumour markers in carcinoma of the cervix treated more consistently, predominantly by radiotherapy alone. In addition, as few studies have evaluated the independence of predicting disease outcome after allowing for stage of disease (Åvall-Lundqvist *et al.*, 1992), multivariate analyses were carried out to determine the independence of serum marker prognosis.

### Materials and methods

### *Patients*

Peripheral blood samples were obtained from patients who had been referred to the Christie Hospital for treatment for invasive primary carcinoma of the uterine cervix between June 1990 and September 1993. Informed consent was obtained from all patients. During examination under anaesthetic, a biopsy of the tumour was taken for independent assessment of diagnosis and histological grading. Clinical staging, according to the FIGO classification, of the patients was performed during the examination. All the patients were treated according to strict protocols. Only patients receiving radical treatment (i.e. with curative intent) were included in the analyses with patient outcome. The majority of these received radiotherapy alone as described previously (West *et al.*, 1993). A small number were treated with curative intent with radiotherapy plus surgery (4) or chemotherapy (11). Twenty patients received palliative radiotherapy only and treatment follow-up was not obtained for 20 patients.

### *Serum marker analysis*

Peripheral blood samples were taken from the patients on the day before treatment with radiotherapy. These were separated as described elsewhere (Elyan *et al.*, 1993), coded and the serum stored at $-80°C$. All analyses were performed blind. The assays were carried out using commercially available kits. CA125 radioimmunoassay kits were purchased from CIS (UK) (High Wycombe, UK). SCC concentrations were estimated using the Abbott SCC RIABEAD (Abbott Laboratories, Chicago, Il, USA) and TPA concentrations using PROLIFIGEN TPA IRMA (AB Sangtec Medical, Bromma, Sweden). The manufacturer's recommended procedures were followed throughout.

### *Data analysis*

As values for all three serum markers did not appear to be normally distributed, the Mann–Whitney *U*-test was used to test for the level of significance of differences between data sets. Regression analysis was used to look for correlations between factors. The probabilities of overall patient survival, locoregional control and metastasis-free survival were determined using log-rank analysis, with the continuous variables grouped into two bands, either above and below the median values or using a cut-off defined as the top of the normal range (as commonly used in the literature). Multivariate Cox analysis was used to test for independence from stage. A significance level of 0.05 was used throughout.

### Results

Data are summarised in Tables I–III. Mean values with standard deviations for CA125, SCC and TPA were $37 \pm 86$ U ml$^{-1}$, $10 \pm 19$ ng ml$^{-1}$ and $127 \pm 163$ U ml$^{-1}$ respectively. A check was made of the reproducibility of the three diagnostic tests. Triplicate analyses carried out on 40 samples yielded a mean coefficient of variation (CV) of 8% intra-assay variability for all three markers. This experimental error was considerably smaller than the inter-individual variability, which gave CVs of 232%, 190% and 128% for CA125, SCC and TPA respectively.

Correspondence: C West
Received 25 October 1994; revised 30 May 1995; accepted 11 July 1995

**Table I** Serum CA125 levels in relation to disease stage

| *Stage of disease* | n | *Mean ± s.d. (U ml⁻¹)* | *Median (U ml⁻¹)* | *Range (U ml⁻¹)* | P* | *Elevated[a] levels (%)* |
|---|---|---|---|---|---|---|
| All | 158 | 37 ± 86 | 17 | 0–734 | | 22 |
| I | 41 | 17 ± 16 | 12 | 0–69 | | 9 |
| II | 56 | 26 ± 20 | 17 | 3–175 | 0.035 | 19 |
| III | 48 | 68 ± 43 | 23 | 4–734 | 0.002 | 35 |
| IV | 10 | 38 ± 27 | 20 | 10–154 | 0.085 | 25 |

*Denotes significance for differences between stage I and other disease stages. [a]The percentage of patients with marker levels above 35 U $ml^{-1}$. II *vs* III $P=0.09$; II *vs* IV $P=0.49$; III *vs* IV $P=0.89$.

**Table II** Serum SCC levels in relation to disease stage

| *Stage of disease* | n | *Mean ± s.d. (ng ml⁻¹)* | *Median (ng ml⁻¹)* | *Range (ng ml⁻¹)* | P* | *Elevated[a] levels (%)* |
|---|---|---|---|---|---|---|
| All | 166 | 10 ± 19 | 3.0 | 0–140 | | 51 |
| I | 46 | 3.8 ± 2.0 | 0.6 | 0–64.9 | | 22 |
| II | 59 | 5.8 ± 8.2 | 3.0 | 0–41.0 | 0.0007 | 54 |
| III | 48 | 18.0 ± 26.8 | 7.6 | 0.2–140.2 | 0.0001 | 76 |
| IV | 10 | 24.1 ± 19.8 | 13.2 | 0.3–82.5 | 0.0011 | 88 |

*Denotes significance for differences between stage I and other disease stages. [a]The percentage of patients with marker levels above 2.5 ng $ml^{-1}$. II *vs* III $P=0.0003$; II *vs* IV $P=0.012$; III *vs* IV $P=0.51$.

**Table III** Serum TPA levels in relation to disease stage

| *Stage of disease* | n | *Mean ± s.d. (U ml⁻¹)* | *Median (U ml⁻¹)* | *Range (U ml⁻¹)* | P* | *Elevated[a] levels (%)* |
|---|---|---|---|---|---|---|
| All | 166 | 127 ± 163 | 69 | 14–1145 | | 37 |
| I | 41 | 17 ± 16 | 12 | 15–1145 | | 21 |
| II | 56 | 26 ± 20 | 17 | 14–388 | 0.019 | 41 |
| III | 48 | 68 ± 43 | 23 | 24–1132 | 0.001 | 55 |
| IV | 10 | 38 ± 27 | 20 | 32–445 | 0.007 | 63 |

*Denotes significance for differences between stage I and other disease stages. [a]The percentage of patients with marker levels above 100 U $ml^{-1}$. II *vs* III $P=0.004$; II *vs* IV $P=0.097$; III *vs* IV $P=0.96$.

**Table IV** Serum marker levels in relation to outcome following treatment by radiotherapy

| | *Status* | n | *Mean ± s.d.* | *Median* | *Range* | P |
|---|---|---|---|---|---|---|
| CA125 (U ml⁻¹) | A | 92 | 23 ± 25 | 15 | 4–175 | |
| | D | 27 | 97 ± 175 | 30 | 3–620 | 0.015 |
| SCC (ng ml⁻¹) | A | 95 | 7 ± 14 | 2 | 0–109 | |
| | D | 28 | 23 ± 32 | 10 | 0–140 | 0.006 |
| TPA (U ml⁻¹) | A | 95 | 97 ± 99 | 66 | 14–595 | |
| | D | 28 | 186 ± 177 | 113 | 22–683 | 0.015 |

Patients were either alive and well (A) or dead of disease (D). The follow-up times ranged from 1 to 4 years.

Weak, but significant, correlations were seen between the levels of the different serum markers. There were, however, no significant associations between marker levels and either patient age, disease grade or tumour volume. The levels of SCC were significantly higher in 120 squamous cells tumours (11.8 ± 20.8 ng $ml^{-1}$) compared with ten adenocarcinomas (0.7 ± 1.0 ng $ml^{-1}$). No differences between tumour types were seen for either CA125 or TPA. For all serum markers, levels increased with increasing disease stage (Tables I–III).

A comparison was made between the pretreatment serum marker levels found in patients who were either alive and well, or dead of disease following radiotherapy (1 year minimum follow-up time). Marker levels were significantly higher in the dead outcome group for all three markers studies (Table IV). A comparison was also made between the pretreatment serum marker levels found in patients in relation to the development of metastatic disease. Patients who developed metastatic disease had significantly higher pretreatment serum marker levels (Table V).

Using log-rank analysis and stratifying according to median values (the most objective criteria to use), a study was made of treatment outcome in terms of either survival (Figure 1), local control (Figure 2) or metastasis-free survival (Figure 3). For comparison, the prognostic ability of disease stage is illustrated for the same series of patients. Survival levels were significantly higher for patients with low (below the median) serum marker levels for all three markers studied. Serum marker levels were poorer at predicting local control levels compared with metastasis-free survival. A significant difference in local control was only seen for CA125 (Figure 2). However, significant differences in metastasis-free survival were seen for all tumour markers (Figure 3). For comparative purposes the data were also analysed using the manufacturer's recommended cut-off levels i.e. 2.5 ng $ml^{-1}$ for SCC, 35 U $ml^{-1}$ for CA125 and 100 U $ml^{-1}$ for TPA. These cut-off levels are commonly used in the literature. Although the higher cut-off gave slightly better discrimination, similar results were obtained when the data were stratified by the two different methods (Table VI).

An investigation was made of the independence of the

**Table V** Serum marker levels in relation to the development of metastatic disease following treatment with radiotherapy

| | Status | n | Mean ± s.d. | Median | Range | P |
|---|---|---|---|---|---|---|
| CA125 (U $ml^{-1}$) | N | 97 | 34 ± 89 | 16 | 4–620 | |
| | M | 23 | 64 ± 95 | 41 | 3–459 | 0.003 |
| SCC (ng $ml^{-1}$) | N | 102 | 8 ± 16 | 2 | 0–109 | |
| | M | 22 | 20 ± 33 | 10 | 0–140 | 0.004 |
| TPA (U $ml^{-1}$) | N | 102 | 102 ± 106 | 67 | 14–595 | |
| | M | 22 | 192 ± 179 | 119 | 24–683 | 0.009 |

Patients were either metastasis-free (N) or with metastases (M). The follow-up times ranged from 1 to 4 years.

**Figure 1** Patient survival in relation to disease stage and serum marker levels following treatment by radiotherapy. The numbers of individuals studied were 125, 119, 123 and 123 for stage, CA125, SCC and TPA respectively. Follow-up times ranged from 1 to 4 years.

**Figure 2** Local control in relation to disease stage and serum marker levels following radical treatment by radiotherapy. The numbers of individuals studied were 125, 119, 123 and 123 for stage, CA125, SCC and TPA respectively. Follow-up times ranged from 1 to 4 years.

**Figure 3** Metastasis-free survival in relation to disease stage and serum marker levels following radical treatment by radiotherapy. The numbers of individuals studied were 125, 119, 123 and 123 for stage, CA125, SCC and TPA respectively. Follow-up times ranged from 1 to 4 years.

**Table VI** Log-rank analyses showing the probability of a pretreatment variable to predict treatment outcome in terms of either overall survival, local control and metastasis-free survival

| *Variable* | *Cut-off* | *Survival* | *Local control* | *Metastases* |
|---|---|---|---|---|
| Stage | | <0.001 | <0.001 | <0.001 |
| TPA | 69 U ml$^{-1}$ | 0.024 | 0.64 | 0.02 |
| | 100 U ml$^{-1}$ | 0.005 | 0.42 | 0.027 |
| SCC | 3.0 ng ml$^{-1}$ | 0.017 | 0.065 | 0.007 |
| | 2.5 ng ml$^{-1}$ | 0.005 | 0.018 | 0.001 |
| CA125 | 17 U ml$^{-1}$ | 0.014 | 0.013 | 0.041 |
| | 35 U ml$^{-1}$ | <0.001 | 0.006 | <0.001 |

Patients were stratified according to either the median serum marker levels (upper values) or the manufacturer's recommended cut-off levels (lower values). All numbers given are *P*-values.

prognostic ability of serum marker measurements in relation to disease stage (Table VII). Using Cox multivariate analysis disease stage was the most important prognostic variable. After allowing for stage, only CA125 was a significant independent predictor of treatment outcome. Similar results were obtained when the data were analysed using two different cut-off levels.

### Discussion

The serum marker levels found in this study were similar to those reported elsewhere. For example in 142 patients with carcinoma of the uterine cervix. Åvall-Lundqvist *et al.* (1992) reported median values of 21 U ml$^{-1}$, 2 ng ml$^{-1}$ and 50 U ml$^{-1}$ for CA125, SCC and TPA respectively. Increasing marker levels with disease stage has also been described by others (e.g. Brioschi *et al.*, 1991; Åvall-Lundqvist *et al.*, 1992).

Using the manufacturer's recommended cut-off levels of 2.5 ng ml$^{-1}$ for SCC, 35 U ml$^{-1}$ for CA125 and 100 U ml$^{-1}$ for TPA, elevated levels were seen in 51%, 22% and 37% of patients for SCC, CA125 and TPA respectively (Tables I–III). These values are within the ranges reported by others using the same cut-off levels e.g. 44–57% for SCC (Duk *et al.*, 1990; Åvall-Lundqvist *et al.*, 1992), 23% for CA125 (Gadducci *et al.*, 1990; Åvall-Lundqvist *et al.*, 1992) and 28–47% for TPA (Åvall-Lundqvist *et al.*, 1992; Ngan *et al.*, 1994).

Mean serum concentrations of SCC were 7 and 23 ng ml$^{-1}$ for patients free and dead of disease respectively. These values are similar to values reported elsewhere of 6 ng ml$^{-1}$ for patients with no evidence of disease and 16 ng ml$^{-1}$ for those with recurrent disease (Maiman *et al.*; 1989). This study has also confirmed that pretreatment measurements of the three markers can predict treatment outcome in carcinoma of the cervix. This has been reported for CA125 (Duk *et al.*, 1990; Åvall-Lundqvist *et al.*, 1992), SCC (Maiman *et al.*, 1989; Duk *et al.*, 1990; Ngan *et al.*, 1994) and TPA (Ngan *et al.*, 1994). The larger analysis described here, however, is the first to show that CA125 is better at predicting recurrence-free survival whereas SCC and TPA predict for metastasis-free survival. In addition, studies published previously on cervical carcinoma involved treatment predominantly with surgery plus radiotherapy. Therefore, by confirming the prognostic significance of serum marker measurements for patients treated with radiotherapy alone, this study illustrates the independence of the findings from mode of treatment.

As reported here and elsewhere serum marker concentrations show a strong stage dependence. It is, therefore, not surprising to find that after allowing for stage the prognostic significance of all three markers was either lost or reduced. Only CA125 was shown to be an independent indicator of prognosis. This finding contrasts with the results of Åvall-Lundqvist *et al.* (1992) who showed that both CA125 and SCC were significantly related to survival, in addition to stage. Similar levels of adenocarcinomas and squamous cell carcinomas, and similar follow-up times were found in both studies. Therefore, the disparate findings may be related to either differences in treatment methods, i.e. radiotherapy alone (this study) or predominantly surgery plus radiotherapy (Åvall-Lundqvist *et al.*, 1992) or differences in the

**Table VII** Cox multivariate analyses showing the relative risk (RR) for survival, local recurrence-free survival and metastasis-free survival

| | *Survival* | | *Local control* | | *Metastasis-free* | |
|---|---|---|---|---|---|---|
| *Variable* | *RR* | P | *RR* | P | *RR* | P |
| Stage *vs* I | | | | | | |
| II | 2.5 | <0.001 | 2.1 | 0.005 | 3.7 | 0.001 |
| III | 6.9 | | 6.0 | | 5.3 | |
| IV | 15.2 | | 9.1 | | 22.9 | |
| CA125 | 2.3 | 0.041 | 2.0 | 0.017 | NS | |
| Stage *vs* I | | | | | | |
| II | 2.6 | 0.003 | 2.4 | 0.002 | 3.1 | 0.005 |
| III | 5.9 | | 6.9 | | 2.9 | |
| IV | 11.4 | | 10.4 | | 18.3 | |
| CA125 | 3.1 | 0.009 | NS | | 8.4 | <0.001 |

Both the median (upper half) and the top of the normal range (lower half) were used as cut-off values.

division of patients between disease stages with 28%, 36% and 29% of patients having stage I, II and III disease (this study) compared with 50%, 25% and 19% of patients having stage I, II and III disease (Åvall-Lundqvist *et al.*, 1992).

Previous work by us has shown that measurements of tumour intrinsic radiosensitivity (assessed as surviving fraction at 2 Gy, $SF_2$) are a good predictor of local control for carcinoma of the cervix treated with radiotherapy (West *et al.*, 1993). It will be of interest in the future to combine the radiosensitivity data with that for the serum markers in particular to investigate whether $SF_2$ and CA125 might combine to give a better prediction of locoregional failure than either alone. There is some preliminary evidence to suggest that $SF_2$ and the serum markers are independent parameters. For 53 of the tumours studied in this work, $SF_2$ values were obtained. There were no correlations between $SF_2$ values and any of the marker levels ($P>0.20$ for all). Tumour radiosensitivity data are still being accrued and a multivariate analysis will be carried out in the future.

In summary, this work has confirmed the prognostic significance of pretreatment measurements of serum markers in patients with cancer of the cervix. However, the study has shown that, after allowing for stage, their influence on treatment outcome is either lost or reduced and only CA125 appears to have any value as a pretreatment prognostic variable. In the future it will be of interest to evaluate CA125 alongside measurements of tumour intrinsic radiosensitivity. This will determine whether combinations of $SF_2$ and CA125 can improve the prediction of treatment outcome already reported for $SF_2$ alone in carcinoma of the cervix undergoing radiotherapy.

**Acknowledgements**
This work was supported by the Cancer Research Campaign and the Christie Hospital Endowment Fund.

**References**

ÅVALL-LUNDQVIST E, NORDSTRÖM L, SJÖVALL K AND ENEROTH P. (1989). Evaluation of seven different tumour markers for the establishment of tumour marker panels in gynecologic malignancies. *Eur. J. Gynaec. Oncol.*, **10**, 395–405.

ÅVALL-LUNDQVIST EH, SJÖVALL K, NILSSON BR AND ENEROTH PHE. (1992). Prognostic significance of pretreatment serum levels of squamous cell carcinoma antigen and CA 125 in cervical carcinoma. *Eur. J. Cancer*, **28A**, 1695–1702.

BRIOSCHI PA, BISCHOF P, DELAFOSSE C AND KRAUER F. (1991). Squamous-cell carcinoma antigen (SCC-A) values related to clinical outcome of pre-invasive and invasive cervical carcinoma. *Int. J. Cancer*, **47**, 376–379.

DUK JM, AALDERS JG, FLEUREN GJ, KRANS M AND DE BRUIJN HWA. (1989). Tumor markers CA 125, squamous cell carcinoma antigen, and carcinoembryonic antigen in patients with adenocarcinoma of the uterine cervix. *Obstet. Gynecol.*, **73**, 661–668.

DUK JM, DE BRUIJN HWA, GROEPIER KH, FLEUREN GJ AND AALDERS JG. (1990). Prognostic significance of pretreatment serum CA 125, squamous cell carcinoma antigen, and carcinoembryonic antigen levels in relation to clinical histopathologic tumor characteristics. *Cancer*, **65**, 1830–1837.

ELYAN SAG, WEST CML, ROBERTS SA AND HUNTER RD. (1993). Use of low-dose rate irradiation to measure the intrinsic radiosensitivity of human T-lymphocytes. *Int. J. Radiat. Biol.*, **64**, 375–383.

GADDUCCI A, FERDEGHINI M, PRONTERA C, GIORDANO M, CRISTOFANI P, BIANCHI R AND FIORETTI P. (1990). A comparison of pretreatment serum levels of four tumor markers in patients with endometrial and cervical carcinoma. *Eur. J. Gynaecol. Oncol.*, **11**, 283–288.

KATO H AND TORIGOE T. (1977). Radioimmunoassay for tumor antigen of human cervical squamous cell carcinoma. *Cancer*, **40**, 1621–1628.

MAIMAN M, FEUER G, FRUCTER RG, SHAW N AND BOYCE J. (1989). Value of squamous cell carcinoma antigen levels in invasive cervical carcinoma. *Gynecol. Oncol.*, **34**, 312–316.

NGAN HYS, CHENG GTS, YEUNG WSB, WONG YLC AND MA HK. (1994). The prognostic value of TPA and SCC in squamous cell carcinoma of the cervix. *Gynecol. Oncol.*, **52**, 63–68.

WEST CML, DAVIDSON SE, ROBERTS SA AND HUNTER RD. (1993). Intrinsic radiosensitivity and prediction of patient response to radiotherapy for carcinoma of the cervix. *Br. J. Cancer*, **68**, 819–823.